# The Bedding and Linen Department—IV

**Published:** 1908-05-23

**Authors:** 


### Nursing Administration.

### THE BEDDING AND LINEN DEPARTMENT.

### IV.—ORGANISATION IN THE WARD.

It will be seen from the inventories already published of the ward equipment of linen and other articles required to be kept in stock that the secret of good organisation in this department lies in arriving at a clear understanding of all probable needs. The matron who desires the machinery to run easily must not spare the oil of patient investigation into the minutest duties which have to be performed. She must provide her workers with the implements needed, not grudgingly, as though it were a crime to require cloths for certain processes, but with the full sense before her of the importance of all these little things, concentrating her full attention on the matter in hand with something of the scientist's spirit.

In large and complex clinical hospitals the inventory is certain to vary with each ward. In more regularly constructed hospitals of the ordinary provincial type the same quantities will be required in each ward, with certain additions as for surgical, gynæcological, and children's wards. It should be the first duty of the matron in undertaking reforms, whether in a new post or in her own hospital, to consult with each ward sister as to the revision of her inventory, and leave no single duty unaccounted for, no vague rags to be summoned on emergency from unseen receptacles, but everything regular, above-board and sanitary. Each ward will need to be treated as a separate unit for purposes of this description, each operating theatre and out-patient or casualty department counting as a ward, with separate inventory.

Having once decided what is required, a matter which experience only can satisfactorily settle, and which may need extensive revision, the next important consideration is the receptacle in which the ward sister may store her supplies. In old-fashioned hospitals it is not uncommon to find that the architect has done his best to make proper organisation in the linen department impracticable by providing no space whatever for the ward supplies of linen. The linen department was perhaps not a conspicuous feature of hospital economy in days of old, and for the central linen-room, where mending and making were carried on and where all supplies were kept, a part of the basement was assigned with such light as was obtainable. No difficulties are insurmountable by an able matron, and good linen departments have been controlled even under these drawbacks. But to ordinary people it is not possible to obtain the best results without proper appliances. And managers who grudge the money for the alterations which would give each ward its own distinct linen department have little conception of the waste of time and energy, the want of control leading to loss and waste, involved in attempting to cope with the varied requirements of every part of the hospital without proper receptacles.

Another great defect, too, inseparable from the central linen room is that the ward sister cannot never get a complete grip over what is used in her own sphere. She loses a valuable part of her training, and whether she is careless or precise can never be known, for she has no opportunity for displaying her talents in this important direction. There are few hospitals in which it would not be possible to fit a spacious linen cupboard outside each ward, so arranged that the shelves are easily accessible, well lighted and planned with special attention to the class of articles to be stored on them. We are confident that expenditure on this necessary part of the ward equipment would repay itself within a short time by the increased hold it would give over the expensive outlay on linen and laundry.

Having planned out a list of the articles needed in each ward, and established in close proximity convenient headquarters for such things as are not in use, the next consideration is to maintain the stock in good working order. To this end an annual stock-taking is usually found sufficient, for when the linen in each ward is renewed, repaired and set in complete order it may be reckoned that it will last a year before it needs serious attention again. The occasion chosen must be the annual cleaning, when, the ward being closed, all the articles can be gathered together and gone through item by item with the ward sister in whose charge they lie. It is more satisfactory if the matron herself can assist at this review, but where this is not practicable the linen sister or housekeeper takes her place. Condemned articles are removed to be turned to account in the linen room in other ways. A note is made of whatever has been lost, and fresh articles are issued to make the number up to stock. The following form* in use at King's College Hospital is an excellent one for the matron's linen register:—

Number of Beds 30.

| On Inventory | Articles | Good | Condemned | Lost | Issued | Total |
|---|---|---|---|---|---|---|
| 72 | Counterpanes ... | 68 | 3 | 1 | 4 | 72 |
| 140 | Blankets ... ... | 125 | 15 | — | 15 | 140 |
| 180 | Sheets ... ... | 167 | 10 | 3 | 13 | 180 |
| 180 | Draw sheets... ... | 143 | 23 | 7 | 30 | 180 |
| 180 | Pillow cases... ... | 172 | 8 | — | 8 | 180 |
| | etc. etc. | | | | | |

At St. Thomas's Hospital as in many others an inventory is taken of the ward linen on the occasion of any change of sisters in the ward. Although the regular renewal of articles takes place annually the sister will on occasions be compelled to requisition supplies to eke out her stock from the central linen store, in which a surplus of everything used in the hospital is kept in readiness for emergencies.

* As supplied by the Scientific Press, 28 Southampton Street, Strand, London, W.C. Price 7s. 6d.

